# Development of a work-integrated learning programme for chronic pain physiotherapy in Dutch private practice using co-design methods: description of a journey

**DOI:** 10.1136/bmjopen-2024-098115

**Published:** 2025-09-21

**Authors:** Han van Dijk, Albère Köke, Stefan Elbers, Christa van Gessel, Rosa de Vries, Ilya Zitter, Rob Smeets, Harriët Wittink

**Affiliations:** 1 Institute for Human Movement Studies, HU University of Applied Sciences Utrecht, Utrecht, The Netherlands; 2 Department of Rehabilitation Medicine, Research School for Public Health and Primary Care CAPHRI, Maastricht University, Maastricht, The Netherlands; 3 Research Group Vocational Education, Research Centre for Learning and Innovation, HU University of Applied Sciences Utrecht, Utrecht, The Netherlands; 4 Centre of Expertise in Rehabilitation and Audiology, Adelante, Hoensbroek, The Netherlands; 5 School of Physiotherapy, Zuyd University of Applied Sciences, Heerlen, The Netherlands; 6 Pain in Motion International Research Group (PiM), Brussels, Belgium; 7 Research Group Innovation of Human Movement Care, Research Centre for Healthy and Sustainable Living, HU University of Applied Sciences Utrecht, Utrecht, The Netherlands; 8 Research Group Co-Design, Research Centre for Learning and Innovation, HU University of Applied Sciences Utrecht, Utrecht, The Netherlands; 9 CIR Clinics in Revalidatie, Eindhoven, The Netherlands; 10 Research Group Lifestyle and Health, Research Centre for Healthy and Sustainable Living, HU University of Applied Sciences Utrecht, Utrecht, The Netherlands

**Keywords:** chronic pain, physiotherapy, implementation, biopsychosocial model, workplace learning, co-design methods

## Abstract

**Background:**

Physiotherapists recognise the biopsychosocial model as important in treating chronic pain. However, the adoption of this model in Dutch private practice is limited. Participatory action research, including co-design methods and an explicit perspective on workplace learning, may be helpful in developing a work-integrated learning programme to facilitate the use of a biopsychosocial perspective in private practice physiotherapy.

**Objective:**

To give insight into the development of a work-integrated learning programme for private practice physiotherapists in assessing and treating patients with chronic pain from a biopsychosocial perspective.

**Methods:**

An interprofessional development team of designers, developers, educational professionals and researchers engaged in a co-design process together with private practice physiotherapists, experts in chronic pain, patients and other relevant stakeholders. In this design process, the team developed several prototypes and the subsequent work-integrated learning programme during three 2-week design sprints, living-lab tests and validation sessions. All available co-design data were structured and analysed by three researchers, resulting in a plan of requirements as a foundation for the work-integrated learning programme.

**Results:**

The data rendered two specific outcomes: (1) a plan of requirements to be used as an educational foundation for the work-integrated learning programme and (2) several prototypes based on the underlying principles that are used in the development and validation of the work-integrated learning programme.

**Conclusions:**

This study shows how co-design methods can be successfully applied to generate insights and develop interventions that bridge theory and practice for physiotherapists working in private practice. The designed prototypes and subsequent distilled plan of requirements for the development of a work-integrated learning programme offer new opportunities to facilitate the transition to working from a biopsychosocial perspective in private practice physiotherapy.

**Trial registration number:**

RAAK.PUB06.014.

**STRENGTHS AND LIMITATIONS OF THIS STUDY**

- A large and diverse group of contributors and stakeholders participated in the design process, ensuring various perspectives and comprehensive input.
- The use of a sprint method facilitated the rapid development of testable prototypes, thereby preventing the excessive expenditure of time and resources.
- Performing an analysis of the design process to create a plan of requirements supports the creation of, and provides insight into, the development of the final programme.
- The iterative nature of co-design may obscure how decisions were made, making it difficult to trace the rationale behind specific design choices.

## Introduction

 Chronic musculoskeletal pain affects 20–30% of the population,^1 2^ making it a major societal problem with high individual costs.^2^ Treatment guidelines refer to the use of the biopsychosocial (BPS) model as the primary perspective for understanding and managing chronic pain.^3^,^6^ It is acknowledged that physiotherapists are well positioned to provide BPS treatment as first-line clinicians in various healthcare settings.^7^ Although physiotherapists increasingly recognise the importance of a BPS perspective in treating chronic pain,^8^,^12^ they struggle to integrate this perspective when working with patients.^9^,^1113^ The actual adoption of the BPS model, including following the respective guidelines, in private practice is therefore currently minimal.^3^ Physiotherapists often describe a lack of knowledge, skills and attitudes in dealing with the psychological and social factors regarding the multidimensional nature of musculoskeletal chronic pain.^8^,^14^ This is attributed to limited attention to these factors during initial education and post-graduate courses for physiotherapists and a lack of guidance in the workplace.^811^,^14^

Within the body of scientific literature, there is limited information on how to best approach the training of physiotherapists to integrate a BPS perspective.^7^ Reporting is sparse and highly variable.^7^ Often, BPS training is done in the context of a randomised controlled trial that aims to investigate a specific intervention.^15 16^ The primary outcome in these studies is focused on the effectiveness of the intervention, instead of on the learning process of the physiotherapist and whether they were able to deliver the BPS-based intervention appropriately. Most courses take place in a classroom setting, removed from real-world practice, and do not take physiotherapists’ prior knowledge and skills into account. Courses that create changes in the attitude, treatment skills and clinical practice are rare.^7 17 18^ A recent scoping review showed that post-graduate courses that solely lean on informing the physiotherapist rather than guiding and giving continued supervision show little promise.^7^ However, initiatives using ongoing supervision and a more practical approach to learning have potential.^719^,^21^ Nevertheless, there is a lack of information available on how these BPS educational programmes were developed, which educational principles they used, and what professionalisation components were offered.^7^

The need for post-graduate courses, lifelong learning and guidance in supporting patients with chronic pain is widely recognised.^7^,^912^ Challenges in the implementation of a BPS treatment approach encompass more than addressing merely limited knowledge and skills. Confidence, clarity of their own role and negotiating patient expectations while maintaining a good therapeutic relationship are reported barriers.^8 12^ It therefore seems logical to assume that an educational intervention should go beyond acquiring knowledge to influence the practice behaviour of the physiotherapist and thereby improve care for patients with chronic pain. Integrating experiences from the workplace together with knowledge, skills and attitudes is the objective of a work-integrated learning (WIL) programme. According to Co-operative Education and Work-Integrated Learning Canada, a WIL programme is the process of curricular experiential education which formally and intentionally integrates a student’s academic studies within a workplace or practice setting.^22^ Björk and Willermark state that WIL is a popular umbrella term needing clarification.^23^ Therefore, WIL was operationalised in the form of a specific plan of requirements (PoR) which was used as the foundation for the presented WIL programme and made concrete through several prototypes, resulting in a validated programme. The simultaneous learning and working that occurs in such a hybrid learning environment^24^ is expected to be instrumental in facilitating the adoption of a BPS perspective in the practice behaviour of physiotherapists.

The target audience, meaning the learners or participants of the WIL programme, in this context are the physiotherapists working with patients with chronic pain. The Netherlands has a mixed public–private system with mandatory health insurance. Physiotherapists are considered part of the primary care system (in Dutch: ‘eerstelijnszorg’) and typically operate in independently owned practices. These practices, while privately managed, are integrated into the publicly regulated healthcare system through contractual arrangements with health insurers. Of the more than 35,000 physiotherapists, the majority (approximately 20,000–25,000) work in these private practices. Physiotherapists act as first-contact providers, especially for musculoskeletal issues.^25^,^27^ Given their clinical autonomy and the variability in patient presentations, physiotherapists in private practice face distinct challenges that warrant targeted professional development through structured learning programmes. This group of private practice physiotherapists is not homogenous and differs in knowledge, skills, attitudes and experiences depending on duration since graduation, school of education and specialisation (eg, manual therapy or mental health physiotherapy). The process of developing an intervention for such a diverse and large target audience is not straightforward. To meet the various learning needs, different perspectives and expertises are essential when designing an educational innovation.^28^ The viewpoints of stakeholders, target audience (private practice physiotherapists) and experts in chronic pain, as well as in education, are valuable to incorporate in the development of such a learning programme. Designing collaborative solutions, which can include designing new courses, is the purpose of an educational co-design project.^29^ Co-design is more aligned with design-oriented work, including the design and development of solutions in practice.^30 31^ Co-design is defined as the creativity of designers and people not trained in design, working together in the design development process.^32^ Design thinking prioritises the perspective of the target audience, as well as continuous collaboration through co-design between stakeholders, designers, experts and researchers throughout the project.^33^ This approach to the development of an educational intervention is expected to lead to a programme that is suited to the personal needs of the learner and has an impact on clinical practice.

Effective co-design is not without its challenges. To increase the understanding of whether co-design can be successfully applied in the development of interventions in the healthcare domain, more examples of good practice are needed.^33^ Therefore, our objective is to give insight into the development of a WIL programme for Dutch private practice physiotherapists in assessing and treating patients with chronic pain from a BPS perspective.

## Methods

### Study design

A participatory action research approach was used to develop a WIL programme aimed at increasing the application of the BPS model in Dutch private practice physiotherapy. Co-design methods utilising design thinking were applied. The reporting of this study is done following the checklist proposed by the Enhancing the QUAlity and Transparency Of Health Research Network for reporting on health research involving human-centred design: the guideline for REporting Design Research.^34^ See online supplemental table S1 for the completed guideline.

### Setting

This study is part of the PAIN study (Pain therapy in private practice), funded by a grant from SIA (the Netherlands Taskforce for Applied Research, number RAAK.PUB06.014). The primary objective is to increase the application of the BPS model in the clinical practice of Dutch private physiotherapists treating patients with chronic pain. The PAIN project consortium consisted of nine private practices, the HU University of Applied Sciences Utrecht, Maastricht University, a patient organisation, a community of pain professionals and the Dutch Physiotherapy Association (in Dutch: KNGF).

### Participants: project team, critical friends, steering committee and stakeholders

Participants in the co-design process at various stages were purposively recruited through a variety of channels, including through consortium partners, a newsletter and the professional network of the researchers. The aim was to assemble a diverse group of professionals for the project team, along with advisors with specific expertise (referred to as ‘critical friends’), a steering committee and a broader group of stakeholders, including appropriate representation of the target audience (i.e., physiotherapists working in private practice with patients with chronic pain), to provide the necessary multifaceted experience-based feedback. During the design process, participants assumed distinct roles, either as members of the project team, as advisors or as part of the broader stakeholder group. The project team followed the design sprint methodology as described by Knapp.^35^ For a detailed description of the sprints, see the ‘Procedure’ subsection in the Methods section.

#### Project team

Central in the design process, a small, designated group worked intensively on questions related to the overall aim of the PAIN project: how to facilitate the adoption of a BPS perspective in the practice behaviour of physiotherapists? This project team was composed of seven members. During the design sprints, the roles in the project team were divided as follows:

- Designer in the role of main facilitator (1)
- Designer in the role of co-facilitator (1)
- Content experts (3): chronic pain (2) and behavioural change (1)
- Physiotherapy student (1)
- Content expert in role of decision maker: chronic pain and research methods (1)

#### Critical friends

‘Critical friends’ were used to validate and support the design process. These critical friends did not attend the entire sprints but were consulted for their specific expertise at various stages during the development process. Their role was to have a fresh (critical) look and additional expertise on what the project team had been doing. The following experts participated as critical friends:

- Content expert on learning environments in vocational education (including lifelong learning) and workplace learning (1)
- Content expert in co-design (1)
- Designer in the role of prototyper/creative thinker (1)
- Experts in chronic pain and research methods (2)

#### Steering committee

The steering committee, consisting of three experts in research methods and chronic pain, monitored overall progress. One also held the decision maker role during the sprints, and all could function as a critical friend.

#### Stakeholders

Several stakeholders, mostly physiotherapists with or without experience in the field of chronic pain (as representatives of the target audience), fulfilled a crucial role in giving *input* to the project team. The patient association (in Dutch: Pijnpatiënten naar één stem), the Dutch Physiotherapy Association (in Dutch: KNGF), physiotherapy educators and students were also represented as stakeholders. On the first day of the sprint, the stakeholders were individually or collectively interviewed by the project team. On the final sprint day, stakeholders from similar backgrounds were invited to *test* the prototypes. These stakeholders in the role of testers were purposively sampled to generate relevant multifaceted feedback. Some of the stakeholders interviewed on the first sprint day were asked to also test and give feedback on the prototypes.

**Figure 1:**
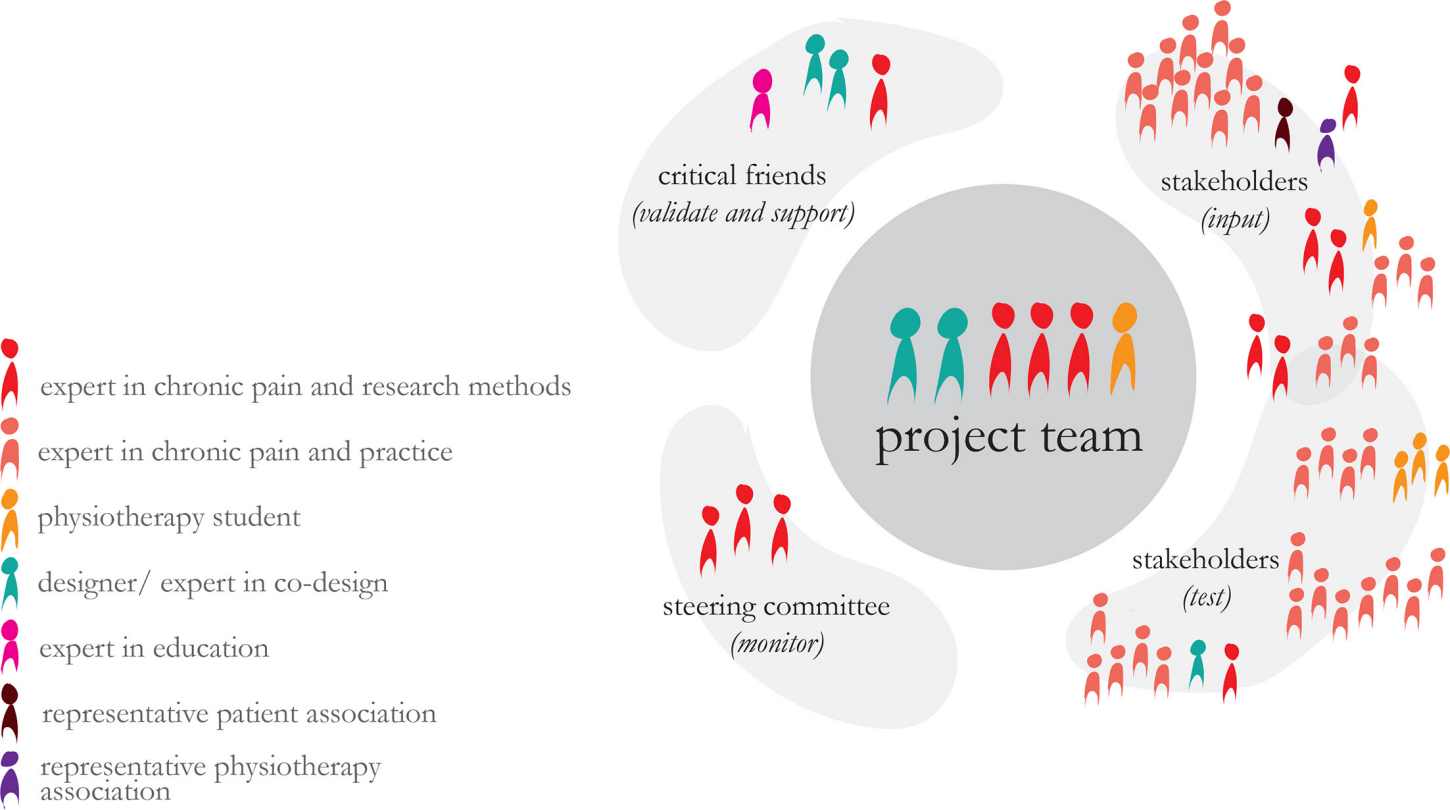
Overview of the different participants in the design process.

**Figure 2:**
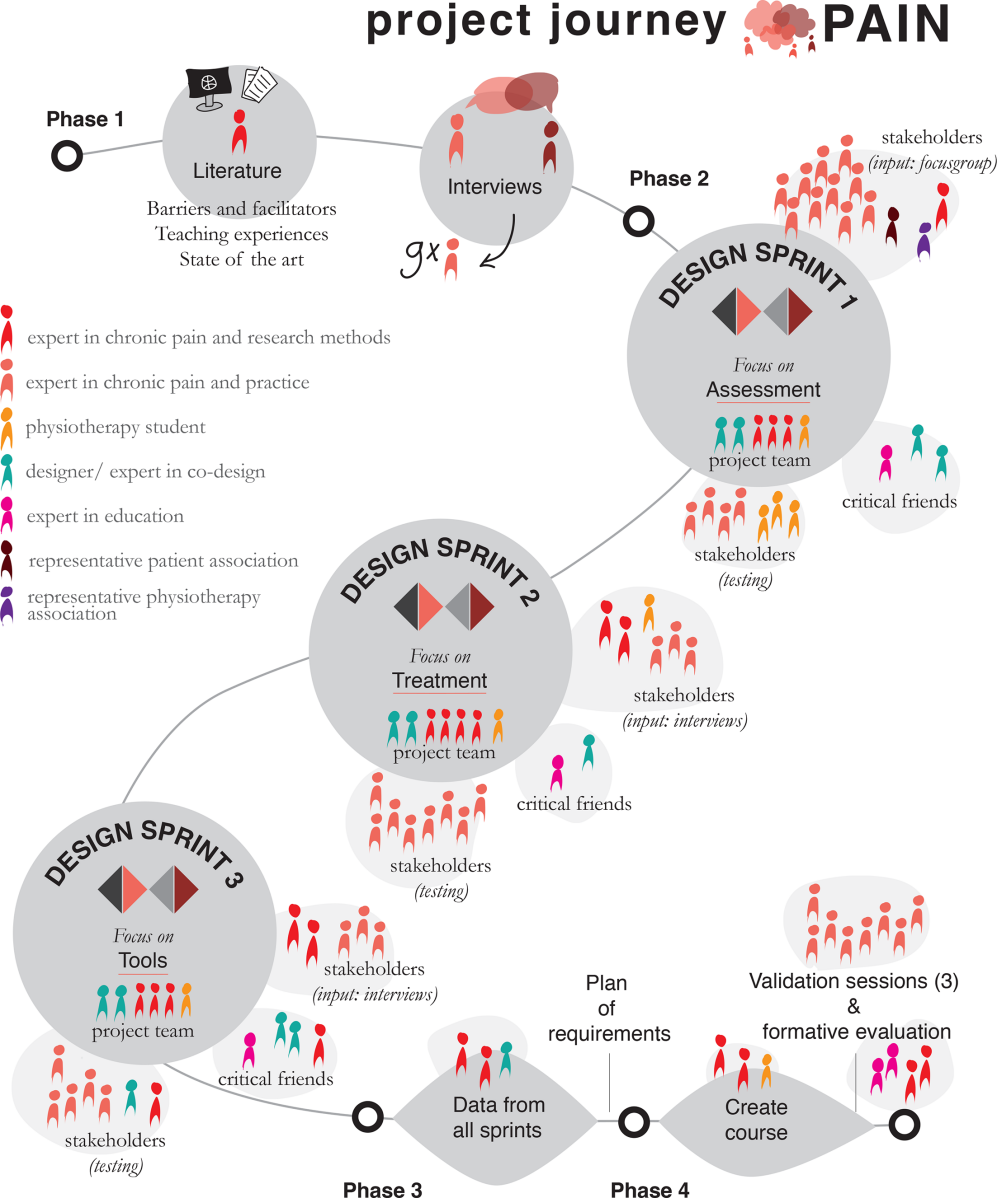
Project journey of the co-design process. The icons represent the correct number of participants to each step and show their background and role in the process.

The different participants are shown in figure 1, and their place in the journey is visualised in figure 2.

### Procedure: project journey

The project journey of the design process of the research project PAIN is shown in figure 2. The journey starts with phase 1, gathering information from the literature and validating this with interviews. This is followed by phase 2, three design sprints focusing on various aspects of clinical practice. The three sprints each had a specific focus. Design sprint 1 focused on patient assessment, design sprint 2 on treatment and design sprint 3 on tools. In this context, ‘a tool’ was defined as an aid to assist the physiotherapist in the application of knowledge and skills and in changing attitude. Thereafter, in phase 3, an analysis of the co-design data was performed to create a clear summary that will assist the final phase of the development of the WIL programme. Finally, in phase 4, the final intervention was developed, including its validation and formative evaluation. Phase 4 will be described in a separate paper.

#### Phase 1: informed intuition

Creating a deep understanding of the physiotherapists for whom the intervention will be designed will positively influence the decision-making process and can be seen as ‘informed intuition.’^36^ To gain a thorough understanding of what is currently known and done, the following actions took place: (1) performing a scoping review on barriers and facilitators to implement a BPS perspective into private practice physiotherapy,^12^ (2) exploring the literature on experiences with educating physiotherapists and (3) establishing the state of the art by studying the physiotherapy pain curricula of the European Pain Federation (EFIC)^37^ and International Association for the Study of Pain (IASP).^38^ Additionally, nine interviews were conducted during this phase with physiotherapists who work in private practice with patients with chronic pain using an interview guide based on the insights obtained from these three actions. Refer to online supplemental table S2 for the interview guide that was used. The aim of these interviews was to validate the information from the scientific literature and gather additional information. They also served to empathise with the stakeholders^39^ and probe the target audience^40^ for further aspects to consider in the next phases of the design process.

#### Phase 2: design sprints

**Figure 3:**
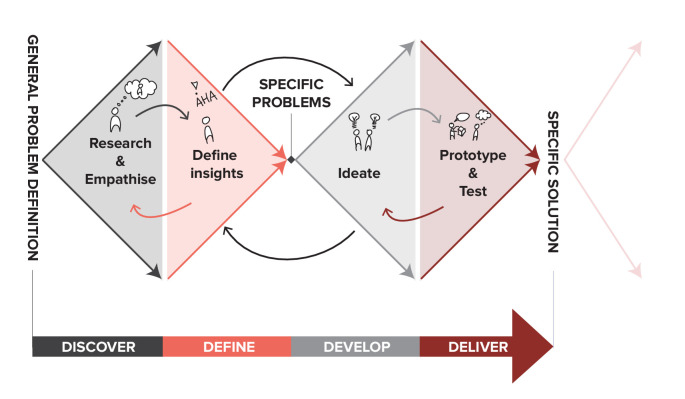
Visual representation of the steps used in design thinking based on the Double Diamond by the Design Council (CC BY 4.0).^41^

The second phase utilised three design sprints. The design sprints were based on the design sprint approach to accelerate problem solving by enabling quick prototyping and testing of potential solutions within a structured 5-day process.^35^ In a sprint, the process of design thinking^39^ is completed in one workweek, aiming to have an evaluated prototype on the final day. By going through a design process quickly, faster choices are forced. As a result, at the end of the week, there is a tangible prototype with which valid feedback can be collected from the target audience, in our case, physiotherapists working in private practice. Studies that apply design thinking work with a multiphase approach that includes versions of the following steps: (1) discover (empathising with stakeholders by conducting focus groups and refining the problem), (2) define (defining the problem), (3) ideate (generating ideas for solutions) and (4 and 5) prototype and test (prototyping and testing the solutions).^41^ A visual representation of these steps is given in figure 3. In participatory action research, inclusion of all relevant parties is realised through active collaboration between stakeholders and researchers, and knowledge is passed along over multiple iterative development cycles,^42 43^ which is in accordance with co-design methods.^30^ The three design sprints each had a different focus and used a multiphase approach described above.

The members of the project team planned each sprint ahead of time based on the information from phase 1 and, for sprints 2 and 3, the previous sprints. This planning consisted of organising the sprint days, informing the participating actors and preparing materials to use during the sprint. Empathising with stakeholders was done by conducting interviews or focus groups at the start of each sprint. These were different from the interviews in phase 1, as they focused on the theme of the sprint, the general problem and possible solutions. The stakeholders were prepared by sending them a sensitiser in advance, either by mail or email. This sensitiser is intended to help them to become more aware of their own experiences, thoughts and feelings related to the topic at hand in preparation for the interview or focus group.^44^ Using qualitative analysis of the data gathered, group discussions within the project team and conversations with the ‘critical friends,’ the main themes were identified and subsequently formulated as ‘how might we’ questions. Underlying principles, helpful models, creative ideas from other fields and viable solutions were subsequently explored with the aim to create testable prototypes. Sprints have an iterative nature, as has the entire co-design process containing the three sprints. Therefore, questions and solutions could build on ideas and material from earlier phases in the process.

All sprints consisted of 4 days (not the traditional 5), and these were spread over 2 weeks (on Tuesdays and Thursdays). This was done to allow the members of the project team to attend to other responsibilities in education and practice since these prevented scheduling a whole week off. Between sprints, preparations for the upcoming sprint were made, and if necessary, further testing of the prototypes was conducted and iterations were developed. The interval between sprints 1 and 2 was 4 weeks, and between sprints 2 and 3, it was 7 weeks. Ideas generated during the sprints, including the feedback received when testing the prototype, were considered during the next sprint.

Initially, when the plan was devised, the sprints would be performed on location (HU University of Applied Sciences Utrecht), but due to COVID-19, the design process took place online. The project team spent about 7.5 hours (including a few breaks) per day working from home. To collaborate, an online whiteboard from the Mural visual collaboration platform was used.^45^

#### Phase 3: analysis and summary of the results

Following the final sprint, two of the principal researchers (SE and HvD), and one of the co-designers (CG), who were all part of the project team during one or more sprints, performed an additional analysis of all the data generated during the co-design process. These data consisted of the filled-out sensitisers, field notes made in the online MURAL whiteboard during the focus groups, interviews, expert sessions and testing, as well as ideas that emerged during the interviews and collaborative sessions. Step by step, they went through interview and focus group data, team discussions, expert opinions given and reflective data gathered during and after sprints. A combination of thematic coding, concept mapping and relational mapping was employed to synthesise these insights into themes.^46 47^ This was done by reading and familiarising with the data, looking at the generated themes and thorough discussion. The main themes were summarised and coupled with supporting models and theory and the proposed solutions that were transformed into prototypes. The summary was subsequently visualised into a PoR as an underlay for the development of the final intervention. The PoR was translated into English by one of the principal researchers (HvD) using feedback from the members of the project team.

## Results

### Phase 1: informed intuition

The actions during the preparatory phase resulted in several perspectives that informed the design process. The scoping review identified barriers and facilitators (including knowledge, skills and attitudes), environmental context and resources, role clarity, confidence, therapeutic alliance and patient expectations.^12^ A study of the literature, confirmed by a scoping review by Simpson *et al*, showed that when post-graduate courses utilise merely didactic interventions focused on improving knowledge, this might not facilitate the change needed.^7^ Experiential learning, longer duration of a course, additional guidance and a personal learning trajectory were all mentioned as faciliatory for learning. These insights were confirmed during interviews with the nine physiotherapists in the Dutch context. The curricula of EFIC^37^ and IASP^38^ served as an evidence-informed framework for the development of the course and were analysed to determine important themes.

The project team took several actions based on the gathered insights. These included constructing topic guides to be used for the focus groups and interviews that took place during the sprints in phase 2, sensitisers that help participants prepare for and reflect on their experiences before engaging in generative sessions,^44^ involving an education and workplace learning expert, operationalising learning outcomes into recognisable activities of daily practice and sharing acquired insights with the project team and other relevant actors during the design process.

### Phase 2: design sprints

#### Phase 2a—sprint 1: assessment

Focus group discussions drew attention to the complexity and variability of assessing patients with chronic pain, as well as aspects of the behaviour change needed in the individual physiotherapist. The project team used Vygotsky’s idea of the zone of proximal development^48^ to help attendees reflect on their current practices and set personal learning goals. The prototype allowed attendees to connect different aspects of their learning to Vygotsky’s model, encouraging them to reflect and apply these insights to real-world situations.

#### Phase 2b—sprint 2: treatment

Topics mentioned during the interviews at the beginning of the second sprint had to do with the attitude and facilitation of a fundamental behaviour change of physiotherapists. Interviewees highlighted the complex nature of dealing with patients with chronic pain. The project team focused on addressing key questions related to complexity, transitioning from a controlled university environment to a real-world clinic, and bridging the gap between understanding and action. They emphasised collecting workplace experiences and facilitating knowledge acquisition, as well as the development of practical skills and appropriate professional attitudes. Prototypes included a diverse course schedule, an onboarding exercise, personas for reflection and a knowledge bank.

#### Phase 2c—sprint 3: tools

The third sprint was intended for the development of tools that facilitate a transition towards working from a BPS perspective. The interviews at the start of this sprint pointed to the importance of embracing the complexity, the usability of the tools, the therapeutic relationship with the patient and the identity of the physiotherapist. The project team focused on designing a personalised tool for BPS assessments in patients. They emphasised the need for creativity, flexibility and communication skills. The prototype included an experience-based workshop integrating functional capacity testing with exposure-based treatment, a card game and a modifiable clinical reasoning template.

The central questions, underlying principles used, and the developed and tested prototypes for each sprint are shown in table 1.

**Table 1:** Sprint questions, principles and prototypes for the three sprints

	Generated sprint questions	Underlying principles	Developed and tested prototypes
Assessment	How might we get attendees to let go of what they normally do (shake them up) and build trust at the same time? How might we teach a dynamic skill?	Reflection on own practice. Formulating individual learning goals. Vygotsky’s zone of proximal development.^48^ Getting out of the comfort zone into a space where the need to let go and the motivation to learn are present. Boundary crossing between the work and school settings.^24^ Model of constructed vs realistic learning settings and acquisition vs participation learning processes.^57^	A game where, based on a clinical vignette, aspects of the diagnostic process could be mapped on the circles of Vygotsky, with the objective of facilitating reflection and formulating individual learning goals.
Treatment	How might we offer stability in dealing with complexity? How might we facilitate the transition from a controlled environment to a ‘real’ environment? How might we make the step from ‘understanding something’ to ‘doing something’ or vice versa?	Model of constructed vs realistic learning settings and acquisition vs participation learning processes.^57^ Collecting experiences from the personal workplace of the attendee. Reduce the amount of basic knowledge that needs to be taught in the course and integrate this information. Facilitate boundary crossing between different settings of the learning environment and through a variation of work forms.^24^	A course setup that coupled the content of the activities to a variety of work forms. An onboarding exercise challenging the attendee to reflect on their own practice behaviour before enrolling in the programme. Several personas to enable reflection on the journey through the programme. A knowledge bank to give attendees the opportunity to deepen their understanding on topics as needed.
Tools	How might we design a tool that can be personalised and that facilitates performing a biopsychosocial assessment in a patient.	Aspects like epistemic, spatial, instrumental, temporal and social are valuable to consider when developing the learning environment.^58^ The complexity of chronic pain, the variability of different patients and the need for a dynamic use of skills, calls for tools that assist, stimulate creativity and at the same time give a certain amount of freedom. The importance of communication skills, personalised assessment and treatment and functional capacity testing are seen as valuable. Workplace learning and experience-based learning assist the attendee to learn the attitudes and principles, while finding a personal way of adopting these for their own practice.	An experience-based workshop integrating functional capacity testing with exposure-based treatment. A card game that facilitates the use of functional capacity testing in a creative way. A modifiable clinical reasoning template. A learning journey for the attendees with a storyboard.

### Phase 3: analysis and summary of the results

#### Plan of requirements

**Figure 4:**
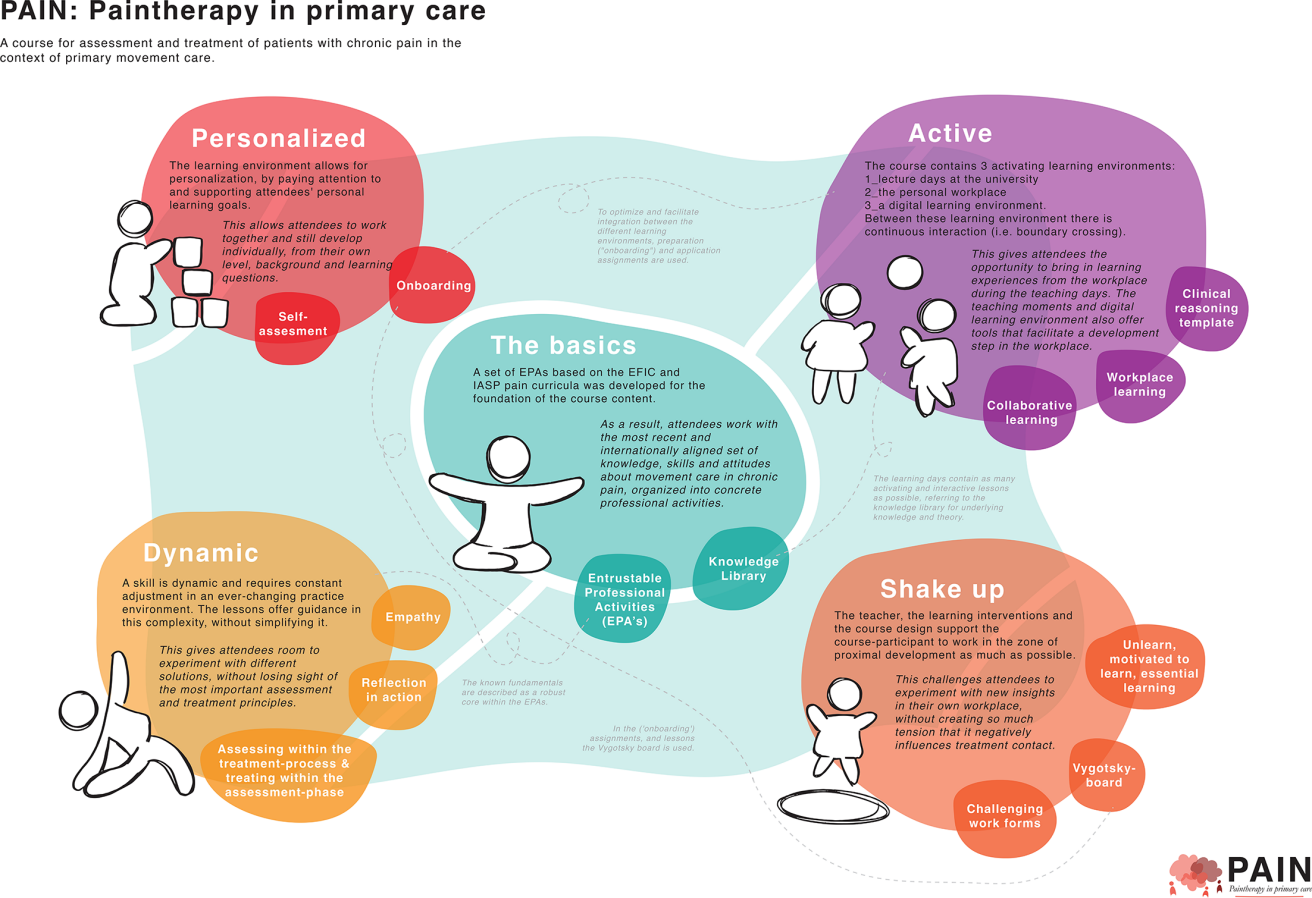
Visual representation of the plan of requirements. EPA, entrustable professional activities; EFIC, European Pain Federation; IASP, International Association for the Study of Pain.

Several themes were consistently found and deemed important throughout the entire co-design process. These five overarching insights were summarised in a PoR, which would subsequently function as an underlay for further development of the course. In the PoR, the themes are presented as design principles that serve as a base for the final course development. See the visual representation of the PoR in figure 4. The reverse side of the PoR, which contains a summary of the principles and used prototypes, is presented as online supplemental file 1.

The theme *the basics* refers to the content that needs to be covered in the course. The knowledge, skills and attitudes that the attendees will develop will have to fit within an internationally accepted framework. The theme *personalised* refers to the importance of enabling individualised trajectories in learning. The pre-existing knowledge, skills, attitudes and experiences of attendees can differ substantially. Creating space for personal goals, as well as meeting attendees ‘where they are at,’ ensures value for everyone. The theme *active* refers to the facilitation of active learning in different learning settings. Three learning settings are distinguished: (1) course days at the university, (2) the attendees’ own workplace and (3) a digital environment. Active learning within, and boundary crossing between, these different settings must be invited. The theme *dynamic* refers to the notion that skills are dynamic in nature and require continuous adjustments in a changing practice environment. Learning interventions have to offer support without simplifying the complexity of chronic pain. This can be done by facilitating a space in which attendees can experiment with different solutions within the framework of the main principles. The final theme of *‘shake up’* refers to the need for change. Attendees will have to be made aware of the importance of letting go of certain attitudes and behaviours, as well as experience the necessity for, and be willing to learn new things. In table 2, the steps taken to incorporate these themes into the course design are summarised.

**Table 2:** Summary of the incorporation of themes in the course design

Theme	Incorporation in the course design
The basics	For this course design, **the curricula of EFIC and IASP** were chosen as a knowledge base to ensure that the attendees follow international standards for treating chronic pain. To facilitate the application of knowledge, skills and attitudes to the workplace, the learning outcomes of the two curricula were to be **operationalised** by translating these **into recognisable activities**. Individualised learning settings and further exploration were enabled by utilising a **digital environment** (EFIC Academy^59^). This knowledge bank contains background material in the form of videos, scientific articles and other sources of information.
Personalised	In this course design, an **‘onboarding’ assignment** was used to prime the attendees regarding the content and form of the programme. The assignment consisted of a clinical vignette with accompanying questions to challenge personal reflection, self-assessment and goal setting considering the various topics of the course. By gathering this information ahead of the session, the educator gained insight into the level and focus of each attendee, thereby making **student-focused guidance** possible. In addition, this assignment supports the activation of attendees.
Active	For this course, assignments and work forms were designed with a continuous variation on two dimensions: (1) constructed vs realistic learning settings and (2) acquisition vs participation learning processes to invite attendees to learn from and with each other within all **three learning environments**. This was done by collecting goals for learning based on experiences in the workplace, connecting these to, for example, a knowledge clip or a simulation assignment, and follow-up with an exercise for processing the information in the workplace. A tool was used to support and **guide clinical reasoning** without ignoring the complexity of the patient with chronic pain.
Dynamic	In this course-design, classes contained an **iterative clinical reasoning** process, in which assessment and treatment were integrated and in quick succession. The changeability of factors like coping, resilience, illness perceptions and cognitions on pain could be explored and challenged early in the treatment process. Attention was given to developing the aptitude of empathy since this **dynamic attitude** was deemed crucial for an effective therapeutic alliance. The attendee was also invited and taught to **‘reflect in action’**: reflect on an experience as it is taking place and adjust accordingly.
Shake up	In this course design, the educator, learning interventions and setup of the programme supported the attendee to work in the **zone of proximal development** as much as possible. **Challenging work forms** invited attendees to experiment with new insights and novel ways of working. A **safe learning environment** was curated to prevent a build-up of tension that might hinder experimentation or negatively influence the therapeutic relationship with the patient in the workplace.

## Discussion

### Summary of the results

The study involved a co-design approach to the development of a WIL programme for physiotherapists working in private practice. From this process, five key themes emerged that could guide the development of a course aimed at helping physiotherapists increase the application of a BPS perspective when assessing and treating patients with chronic pain. These themes are ‘the basics,’ ‘personalised,’ ‘active,’ ‘dynamic’ and ‘shake up.’ By incorporating these themes and the evaluated prototypes, the course can be effectively developed.

The project benefitted from contributions by various stakeholders, including the target audience and patients, and experts in chronic pain, education/workplace learning (including lifelong learning) and co-design. The collaboration allowed for the critical analysis of input from the target audience and other stakeholders, leading to novel questions and innovative solutions. Significantly, the active involvement of educational experts during both the initial phase and subsequent stages of the co-design process was pivotal in shaping an innovative postgraduate WIL programme. This was driven by the realisation that adopting the BPS model necessitated not only knowledge acquisition but also the contextualisation of insights into competent workplace behaviour. Notable discoveries through this collaboration were the focus on the facilitation of workplace learning and experiential learning and the importance of personalised learning and reflection. Feedback from the stakeholders on prototypes provided valuable input, for example, on that time is needed at the workplace between course days at the university.

### Comparison with the literature

Patton *et al* (2013 and 2018) investigated undergraduate physiotherapy education and the workplace learning strategies that can be applied to enhance learning.^49 50^ They state that clinical education is a multidimensional learning space that involves workplace factors, professional practices, clinical supervisors’ intentions and students’ dispositions, all interacting to influence and challenge students’ clinical learning.^50^ While the WIL programme developed in this co-design project focuses on post-graduate education, the many factors and the multidimensional learning space described by Patton *et al* are recognisable. In their 2021 scoping review, Simpson *et al*^7^ proposed an adaptation of Daley’s models of learning in continued professional education.^51^ Experiential learning, time between training and learners’ own expectations, among other aspects, are described. These correlate strongly with the themes found in the analysis of our co-design data.

### Strengths and limitations

Several strengths and limitations regarding the study can be identified. A key strength lies in the richness of the participatory action research approach and the inclusive nature of the co-design process. The involvement of a diverse group of stakeholders ensured that multiple perspectives were considered, enhancing the construct validity of the outcomes. This diversity helped align the design with real-world needs of the target audience, increasing the relevance and contextual fit of the results.^52^ The team’s use of informed intuition, supported by a review of the literature, enriched the team’s understanding, fostering empathy with the target audience and preventing redundant steps, thereby contributing to the internal validity.^52^ The iterative sprint methodology allowed for continuous reflection, adaptation and exploration of the problem from multiple angles, supporting process validity through repeated cycles of feedback and refinement. While the shift to online collaboration was initially seen as a barrier, it unexpectedly enhanced transparency by digitally capturing each step of the process. This digital traceability allowed reflective practice and facilitated collaboration, suggesting that remote co-design, when well facilitated, can still yield valid and reliable outcomes. The creation of a PoR provided a structured overview of the long, creative and iterative process, improving transparency and offering a replicable framework for further development.

Despite these strengths, several limitations were identified. Co-design inherently assumes that the necessary expertise is present within the project team. While this can accelerate progress, it introduces risks to validity if the team lacks sufficient diversity or if key members disengage. Such gaps may lead to omissions in the design process, thereby limiting its overall comprehensiveness. Moreover, co-design processes can be ambiguous and non-linear, often lacking clear decision-making structures.^53 54^ This can undermine reliability, as the process may not easily be repeatable or consistently applied across different contexts. We tried to mitigate this by making our team diverse, clearly designating roles, working with critical friends and scheduling probing of the target audience. However, within the project team, the target audience and patients might have been under-represented.

Another limitation of co-design is that it does not typically yield a finished product. Continued development and post-design validation are necessary to ensure that the outcomes are both functional and effective.^55^ Within this study, we tried to account for this by scheduling time for additional development, including continued validation sessions (phase 4). This is, however, outside of the scope of this present study, limiting the presentation of a finished product. Additionally, the involvement of multiple actors increases time and cost, raising questions about the cost-effectiveness of the approach.^56^ Within the PAIN study, deliberate choices were made to mitigate these commonly recognised challenges. Efficiency was enhanced by working within the time constraints of a design sprint and by strategically scheduling stakeholder input to help maintain procedural reliability while preserving the depth of engagement. Despite these efforts, our approach might not be feasible when the aim is to develop cost-effective learning programmes.

This design process adopts a WIL approach, in which the workplace or practice setting plays a central role in the learning process. As the organisational and clinical context of private practice differs from that of the public sector, this WIL programme is specifically designed to meet the needs of physiotherapists working in Dutch private practice settings.

### Next steps and recommendations

The current output of the co-design process, the WIL programme, will need to be refined and validated, employing continued stakeholder engagement. Testing the usability of the programme by pilot implementation with the target audience will increase insights and assist further validation. Follow-up studies into the feasibility of the WIL programme, and the change in practice behaviour physiotherapists show after following the programme (treatment fidelity), will assess sustained impact over time. As the challenges that informed this research are not unique to the studied context of Dutch physiotherapists working in private practice with patients with chronic pain, it can be assumed that insights from this study might extend beyond physiotherapists and the BPS model for chronic pain. By incorporating educational science, our insights can benefit other educational interventions seeking to implement innovative approaches in healthcare. This study can serve as an inspiration for other co-design endeavours. However, rigorous research is necessary to validate these assumptions and explore how these insights can be adapted to other contexts or populations.

## Conclusion

This study aimed to give insight into the development of a WIL programme for private practice physiotherapists in assessing and treating patients with chronic pain from a BPS perspective. The study used a participatory action research approach grounded in co-design principles. A series of structured co-design sprints were conducted, involving a diverse group of stakeholders (eg, educators, students and professionals). The process was iterative and exploratory, combining stakeholder input, literature-informed intuition and creative problem solving. The co-design process rendered two specific outcomes: (1) a PoR to be used as an educational foundation for the WIL programme and (2) several prototypes based on the underlying principles that are used in the development and validation of the WIL programme. This study shows how co-design methods can be successfully applied to generate insights and develop interventions that bridge theory and practice for physiotherapists working in private practice. While the process highlighted the value of inclusive and iterative design, it also revealed challenges related to team composition, decision-making clarity and the need for continued development and validation. The designed prototypes and subsequent distilled PoR for the development of a WIL programme offer new opportunities to facilitate the transition to working from a BPS perspective in private practice physiotherapy.

## Supplementary material

10.1136/bmjopen-2024-098115 online supplemental file 1

10.1136/bmjopen-2024-098115 online supplemental file 2

10.1136/bmjopen-2024-098115 online supplemental file 3

## Data Availability

The raw data supporting the conclusions presented in this study are available on reasonable request from the corresponding author.
