# Supplementary material for: Development of a work-integrated learning programme for chronic pain physiotherapy in Dutch private practice using co-design methods: description of a journey
Source: BMJ Open. 2025 Sep 21;15(9):e098115. doi: 10.1136/bmjopen-2024-098115 (PMC12458634; doi:10.1136/bmjopen-2024-098115)
Supplement: online supplemental file 1 [file bmjopen-15-9-s001.pdf]

**Entrustable Professional Activities (EPA's)**

EPAs are units of professional practice that can be fully entrusted to an individual, once they have demonstrated the necessary competence to execute them unsupervised. In an EPA all the necessary integrated competencies are described that are needed to perform a professional activity (like a patient interview). This enables the translation of knowledge, skills and attitudes from the learning activities directly to a concrete working environment and vice versa.

**Knowledge library**

The digital learning environment comprises a set of important knowledge videoclips, scientific articles, and other sources of information that form the theoretical fundament of the course. This enables tailored development pathways for each individual and provides the opportunity for both deepening and broadening of learning.

**Onboarding**

By using an 'onboarding'-assignment the course-participant is already 'primed' to the content, teaching formats, prerequisite knowledge, and scope of the course. By facilitating a personal perspective on the subject matter as well as individual learning goals, the onboarding assignment helps to relate the course content to the individual context of the course-participant. The teacher can use the information provided in the assignments for his teaching preparations.

**Self-assesment**

The self-assessment  helps both the course-participant and teacher to gain insight in the current proficiency and topics that need attention. These insights help teachers to match the content of their lessons with the skill level of the attendees. An example of a self-assessment is a 'vignette-case' relevant to activities of primary care.

**Collaborative learning**

Within all three learning environments, assignments and work forms are used to invite the attendees to learn with, and from each other.

**Clinical reasoning template**

Attendees are provided with a decision tree to facilitate the clinical reasoning process. This tool helps the course-participant to thoroughly analyze cases without ignoring - or being discouraged by the complexity of all biopsychosocial factors.

**Workplace learning**

During the course  there will be a continuous variation on two levels:
1_constructed versus realistic, and
2_acquisition versus participation.
During the program, the learning environment continuously varies on two dimensions: constructed versus realistic and acquisition versus participation. A typical loop across all quadrants starts by reflecting on workplace situations to set learning goals (realistic), relating these goals to a specific knowledge clip (acquisition) or a simulation exercise (participation) and providing a processing assignment to form intentions on how to implement the newly acquired competence in their own workplace.

**Assessing within the treatment-process & treating within the assessment-phase**

The premise of the course is an iterative clinical reasoning process, in which the changeability of factors such as coping, resilience, illness perceptions, and cognitions about pain, can be assessed and challenged early in the therapeutic process.

**Reflection in action**

The course-participant is supported in working towards reflection in action: reflecting in the moment on an experience or an event, and making adjustments if necessary.

**Empathy**

The skill empathy is crucial for an effective therapeutic alliance. Extra attention is paid to developing this dynamic skill.

**Challenging work forms**

Lessons applying challenging work forms, like 'the goose-cage', are utilized. These lessons aim to challenge the attendees to step outside of their comfort zone, and participate actively in classroom situations and experiential simulations.

**Vygotsky-baord**

The Vygotsky-board challenges the course-participant to formulate personal learning outcomes in the zone of proximal development, defined as the space between what a learner can do without assistance and what a learner can do with guidance or in collaboration with more capable peers.

**Unlearn, motivated to learn, essential learning**

Within the three learning environments attention is paid to
1_unlearning
2_the motivation to learn
3_the necessity to learn.

PAIN
Paintherapy in primary care

**Figure S1** Summary of the principles and used prototypes as shown on the reverse side of the Plan of Requirements (PoR).
