# Supplementary material for: Development of a work-integrated learning programme for chronic pain physiotherapy in Dutch private practice using co-design methods: description of a journey
Source: BMJ Open. 2025 Sep 21;15(9):e098115. doi: 10.1136/bmjopen-2024-098115 (PMC12458634; doi:10.1136/bmjopen-2024-098115)
Supplement: online supplemental file 2 [file bmjopen-15-9-s002.docx]

**SUPPLEMENTARY MATERIAL – Table S1**

Detailed reporting elements following the REDR checklist for reporting health research involving design [1].

| **Item** | | **Paper section** | **Succinct description** | **Included?** | **Where to find in the article** |
| --- | --- | --- | --- | --- | --- |
| 1 | | Title | Title should indicate that the study included a design approach. | Yes | Title |
|  | 1.1 | Abstract | Abstract summarises the salient components, including background, statement of the problem, approach/ methods, findings, results and conclusion. | Yes | Abstract |
| 2 | | Introduction/ background | Overview of the background to the topic, what has been done in the area already and rationale for using the design. | Yes | See below |
|  | 2.1 | Available knowledge | Summary of what is known about the topic and any gaps | Yes | Introduction paragraph 1-3 |
|  | 2.2 | Rationale for design approach | Explanation of design as appropriate to address the topic. | Yes | Introduction paragraph 4 |
|  | 2.3 | Description of design challenge for health | Description of the main research question or health problem that the design-based work aimed to address. | Yes | Introduction paragraph 4 |
|  | 2.4 | Research aims | Overall aims and objectives of the project. | Yes | Introduction paragraph 5 |
| 3 | | Methods/approach | Description of approach used for the research. | Yes | See below |
|  | 3.1 | Theory | Underlying theories that informed this work (if any). | Yes | Methods paragraph 1 (design); paragraph 3 (procedure) with subheadings (preparation, design sprints, and analysis); figure 2. |
|  | 3.2 | Process and timeline | Processes or steps and timeline for the research. | Yes | Methods paragraph 3 (procedure) with subheadings (preparation, design sprints, and analysis); summarized in figure 1. |
|  | 3.3 | Research team characteristics and reflexivity | Individuals involved in the research team and characteristics of these. Ways that reflexivity was addressed. | Yes | Methods paragraph 4 (participants) with subheadings (project team, advisors, steering committee, stakeholders, and consortium); summarized in table 1. |
|  | 3.4 | Site selection | Description of study sites selected. | Yes | Methods paragraph 3 (procedure), subheading phase 2. |
|  | 3.5 | Participant selection and engagement | Process to select participants, description of the participants and  explanation of how they were involved in design. | Yes | Methods paragraph 4 (participants) with subheadings (project team, advisors, steering committee, stakeholders, and consortium); summarized in table 1, visualized in figure 1. |
|  | 3.6 | Ethical considerations | Ethical precautions taken to protect participants, communities, and personal information. | Yes | Methods paragraph 5 (ethical considerations). |
|  | 3.7 | Language | Description of any language considerations. | Yes | Methods paragraph 3 (procedure), subheading phase 3. |
|  | 3.8 | Techniques to understand  (data collection tools and instruments) | Techniques or tools used during the research process. | Yes | Methods paragraph 3 (procedure), subheading phase 2. |
|  | 3.9 | Documentation | Documentation of work undertaken in research. | Yes / No | General description in Methods paragraph 3 (procedure), and Results paragraphs 1, 2, and 3; detailed in table 2 and figure 3. |
|  | 3.10 | Techniques to synthesise | Description of techniques used to synthesise insights, iterate, and analyse data. | Yes | Methods paragraph 3 (procedure). |
|  | 3.11 | Validation approaches | Process of checking that insights, prototypes or other products were validated. | Yes | Methods paragraph 3 (procedure). |
| 4 | | Results/findings from design research and activities | Summary of findings from design activities, resulting insights, what was designed, what resulted from the work and (if available) impact of activities; report on any secondary or ancillary results. | Yes | See below, summarized in Discussion paragraph 1. |
|  | 4.1 | Design research phase | Summary of major insights or reflection from design activities. | Yes | Results paragraph 1, 2, and 3, with further detail in table 2 and figure 3. |
|  | 4.2 | Decision points | Description of decisions made during the design process. | Yes | Results paragraph 1 and 2, with further detail in table 2. |
|  | 4.3 | Evidence of change or impact | Summary of any evidence of change or impact to health or other  facets of the situation. | No | Not within the scope of this article. |
| 5 | | Discussion | Reflection on design as an approach to the health topic and the strengths and limitations of the work. | Yes | Discussion paragraph 1 and 2. |
|  | 5.1 | Design | Reflection of the application of design to this research topic. | Yes | Discussion paragraph 2. |
| 6 | | Conclusion | Implications of this work for the larger field and next steps. | Yes | Discussion paragraph 4. |
| 7 | | Other | Acknowledgements of support,  assistance, funding, statement on conflict of interest and authors’  contributorship. | Yes | Yes |
|  | 7.1 | Glossary | Definition of key terms | No |  |

1 Bazzano AN, Yan SD, Martin J, *et al.* Improving the reporting of health research involving design: A proposed guideline. *BMJ Glob Health*. 2020;5. doi: 10.1136/bmjgh-2019-002248
