# Supplementary material for: Development of a work-integrated learning programme for chronic pain physiotherapy in Dutch private practice using co-design methods: description of a journey
Source: BMJ Open. 2025 Sep 21;15(9):e098115. doi: 10.1136/bmjopen-2024-098115 (PMC12458634; doi:10.1136/bmjopen-2024-098115)
Supplement: online supplemental file 3 [file bmjopen-15-9-s003.docx]

**SUPPLEMENTARY MATERIAL – Table S2**

Interview guide

| Part 1 - Introduction | |
| --- | --- |
| Points of attention   - Working on a relationship with the physiotherapist. The aim is to start a conversation and make the interviewee feel comfortable and zoom in on themes the interviewee likes to talk about. - Use the ‘value exercise’ through The MuralⓇ visual collaboration platform or a physical representation to start the conversation about beliefs and perspective. - Please note that the list of values is a tool and not an end in itself. If a physiotherapist themself has a different value, that is also fine. Be flexible with this. - Note: focus on mapping their beliefs, perspective and views. | |
| In this first part of the interview, we would like to get to know you a little better. We will do this using the following exercise/ questions.  16 values: Health, autonomy, justice, responsibility, financial security, happiness, creativity, expertise, development, resilience, humour, connection, respect, meaning, freedom, control, fun.   - Which of these values is really important to you, as a practitioner? - Which of these values is really important to you, as a person? - Which of these values do you regularly see in patients with chronic pain, during treatments?   [possible follow-up questions]   - What makes [value] important to you? - How do patients see [value] reflected in you? How is [value] expressed? - How did you arrive at [value]? In what ways did [value] become important in your development as a physiotherapist? | |
| Part 2 – Key questions | |
| Goal 1: Mapping learning experiences   - Mapping key steps in development by zooming in on remembered learning experiences and moments that were perspective changing. | |
| [sample questions]   - What is the reason you started studying physiotherapy? - What is your biggest learning moment? | [topics]   - Professional Development - Role of Therapist - Patients - Peer consultation |
| Goal 2: Discussing an ideal situation   - Briefly have an ideal case described. - Ascertain how knowledge and skills contributed to investigate and handle the case. - Gain insight into attitudes (beliefs, feelings, behaviour) towards patients with chronic pain. | |
| [sample questions]   - What tools did you use with this case? - Do you use measurement tools? - Do you use models? | [topics]   - Communication - Screening - Treatment - Biopsychosocial - Patients - Time - Client retention |
| Goal 3: Discuss areas for improvement regarding knowledge and skills in treating patients with chronic pain.   - Have a less ideal situation/ case described. - Ascertain knowledge and skills that the therapist lacked when examining and treating this case. - Gain insight into attitudes (beliefs, feelings, behaviour) towards patients with chronic pain. | |
| [sample questions]   - What did you feel went less well? - What do you think you still needed to help this client successfully? - How do you think you can achieve that? - Do you ever discuss this with colleagues? | [topics]   - Treatment - Communication - Patient - Role of therapist - Time - Client retention |
| Part 3 – closing | |
| Points of attention   - Summarise the conversation. - If necessary, briefly repeat the objectives again. | |
| [sample questions]   - We just discussed 2 different cases. Imagine all your cases were successful, what would you need to get there? - If you get to choose anything, what would you say needs to be in a training programme? - What did you think of the conversation? In addition to what has already been discussed, would you like to add anything or provide any additional explanation? - Would you like to give us anything more about the research project in general, or these interviews in particular?   [possible follow-up questions]   - In the first part, we mainly talked about important moments in your development. Is there anything else you would like to add? - In the second part, we talked about the ideal situation. Is there anything you would like to add? - In the last part, we talked about things you have encountered. Is there anything you would like to add?   [general questions]   - What age are you? - How many hours a week do you practice? - What additional education/courses did you follow? - How many years of work-experience in private practice do you have? - What is your experience in working with chronic pain patients | |
