# Supplementary material for: Development of a work-integrated learning programme for chronic pain physiotherapy in Dutch private practice using co-design methods: description of a journey
Source: BMJ Open. 2025 Sep 21;15(9):e098115. doi: 10.1136/bmjopen-2024-098115 (PMC12458634; doi:10.1136/bmjopen-2024-098115)
Supplement: Supplementary file 4 [file bmjopen-2024-098115.reviewer_comments.pdf]

## PEER REVIEW HISTORY

BMJ Open publishes all reviews undertaken for accepted manuscripts. Reviewers are asked to complete a checklist review form and are provided with free text boxes to elaborate on their assessment. These free text comments are reproduced below.

### ARTICLE DETAILS

**Title (Provisional)**

Development of a Work-integrated Learning Program for Chronic Pain Physiotherapy in Dutch Private Practice using Co-Design Methods: Description of a Journey

**Authors**

van Dijk, Han; Köke, Albère; Elbers, Stefan; van Gessel, Christa; de Vries, Rosa; Zitter, Ilya; Smeets, Rob; Wittink, Harriët

---

### VERSION 1 - REVIEW

---

| | |
|---|---|
| **Reviewer** | **1** |
| **Name** | **French, Helen** |
| **Affiliation** | **Royal College of Surgeons in Ireland, School of Physiotherapy** |
| **Date** | **09-Jun-2025** |
| **COI** | **None** |

---

Well done to the author team on using these methods to develop a learning programme for physiotherapists.

It is well written and presented, but confusing in parts with use of different terminology, or a failure to cohesively link tables or figures to text.

It is not always clear who the key stakeholders are, and despite reference being made to PPI, it is not clear to the reader, where and how they were involved.

As this appears to be focused on physiotherapists in private practice, some context is required in the introduction. Why do they need a learning programme specifically, compared to those working in the public sector?

Further detail, along with other queries/suggestions are provided below

Abstract

Pg 2, line 23. I don't think there is such as word as fundament- please use another word here

Introduction

Pg 3, line 17 change 'attention for these factors' to 'attention to these factors'

Pg 3, line 32 . suggest changing ‘hardly any information’ to ‘a dearth ( or absence or lack) of information’

Pg 3, line 46 What is CEWIL Canada- provide full word for CEWIL

Pg 3. Line 48 –‘Björk & Willermark, 2024’. No need for the year here as you have the numbered citation

Pg 3, line 49 In relation to this statement ‘WIL was unpacked’, not 100% clear here- do you mean it was unpacked by the manuscript authors?

Pg 3, lines 57-59. In relation to this sentence ‘The group of private practice physiotherapists is not homogenous and will differ in knowledge, skills, attitudes, and experiences’. Some more context is required in relation to the target audience for the WIL. Who are the group? Is if physios in specific region, or nationally? Can you provide some details on profile/ numbers etc. Also suggest remove the word ‘will’ (future tense).

Pg 3, lines 60. Can you define who the stakeholders are. By end-users, do you mean people with chronic pain?

Pg 4, lines 5-6. Please provide a definition of ‘co-design’ here, as distinct from Design thinking

Methods

Pg 5, line 7, Was this specifically for private practice physiotherapists in the Netherlands? Please clarify here

Pg 5, line 9 suggest minor rewording to ‘The reporting of this study is in accordance with

the proposed guideline for REporting Design Research’. Why is it a proposed guideline?

Pg 5, line 16. Again, in relation to the larger study, is this specifically targeted towards physiotherapists in the Netherlands? – please clarify

Pg 5, line 21 this subheading is not clear ‘Participants: core group and stakeholders’, as to what is meant by ‘core group’ as not referred to previously. Regarding stakeholders (and further to my previous comment), did this include the end-users.

More clarity is needed in this section regarding the different media used to recruit the different groups, including the ‘experts in chronic pain’ that were mentioned in the introduction.

Pg 5, line 31

The term ‘sprint’ team is used here without out any explanation- please clarify. Suggest explaining earlier in the design section

Description of the various team members is quite confusing to a reader. Whilst table 1 is helpful, it does not really provide information about who occupies these role. Also, not clear where the end-user features

Pg 6, lines 16-27 Procedure

Whilst figure 1 is helpful, suggest alignment with detail in text regarding participants e.g patient representative shown in figure not mentioned in the manuscript. Also, phases are described in text format, but not marked in the figure, so suggest adding in to clearly link the figure with the text.

Pg 6, line 26 does 'last phase' refer to phase 4? – please clarify

Pg 6, line 45- is the interview guide included as a supplementary file? Suggest it should be

Pg 6, line 60- what do you mean by 'potential attendees'?

Pg 7, line 12 – figure 2 does not align with information in text, where phases are described. Can these be merged to show how the Design Thinking elements were incorporated into the different phases described?

Pg 7, lines 54-56. How were interview and focus group data collected? E.g any audio recordings with subsequent transcriptions?

Was a specific analytical framework used? What form of analysis was undertaken e.g. thematic analysis? Suggest supporting reference

Pg 7, line 58- what you mean by 'underlying principles' and provide a supporting reference

Suggest moving the ethical considerations up to the beginning of the methods section e.g. design section.

Results

Pg 8, line 11 delete the year '2021' and change 'focused of' to 'focused on'

Pg 8, lines 38-39. Not 100% clear what this sentence means 'Interviewees called attention…'. Do you mean 'drew attention' or 'highlighted'?

Pg 8, line 44 what do you mean by 'attention to suitable attitudes'?

Table 2: Column 1 Treatment 'How might we offer footing in dealing with complexity' not clear what offer footing means?

Table 2 Colum 3 Tools what do you mean by journey- ie patient journey?

Figure 3 is very well presented and a strong visual. Glossary is very helpful. There are a couple of minor typographical errors: Collaborative learning and Vygotski board. I don't think 'course-participants' should be hyphenated? Is a course participant the same as an attendee as described in table 2? Suggest use of consistent terms

Discussion

Pg 13, line 13- where is the role of the end-user here, suggest they could play an important role e.g. development of personas, clinical vignettes?

Strengths and limitations section- clearly signpost which are the strengths and limitations, Suggest placing this section after the comparison with other literature.

Pg 13, line 36 suggest adding the word ‘process’ after ‘In the co-design sprint’

Pg 13, line 40 regarding this statement ‘Co-design processes can be fuzzy, lacking clarity when it comes to decision-making’, is this your opinion, or can you support with reference?

In the discussion, can the author team outline next steps?

Some discussion is also warranted on the target audience ie ‘physios in private practice’ and why/how this will address their learning needs, and how they differ to those working in the public system.

Was any consideration given to principles of Universal Design for Learning (UDL) https://udlguidelines.cast.org/

Pg 14, lines 16-21.

Separate the recommendations and conclusion section. Not clear what the ‘recommendations’ really refer to. Suggest a more informative subheading.

Conclusion should provide a brief summary of the aims, methods and results. Currently, it does not really conclude the key points.

---

| | |
|---|---|
| **Reviewer** | **2** |
| **Name** | **Kashif, Muhammad** |
| **Affiliation** | **Riphah International University** |
| **Date** | **15-Jun-2025** |
| **COI** | **None** |

---

The study is designed well, therefore few comments for further improvement.

Explain in the details the validity of this program.

Why only private practice was selected.

Describe the in details, what were limitations faced during the study.

Explain and brief the questionnaire used during the study.

Adress the validity and relaibilty of the presented co-design

## VERSION 1 - AUTHOR RESPONSE

| Reviewer: 1 | |
|---|---|
| It is well written and presented, but confusing in parts with use of different terminology, or a failure to cohesively link tables or figures to text. | We have followed your suggestion to align tables and figures consistently with the text, in terminology as well as in structure. |
| It is not always clear who the key stakeholders are, and despite reference being made to PPI, it is not clear to the reader, where and how they were involved. | We have further aligned our terminology and added clarification where needed as per your suggestion. |
| As this appears to be focused on physiotherapists in private practice, some context is required in the introduction. Why do they need a learning programme specifically, compared to those working in the public sector? | We can see how, from a non-Dutch perspective, this may seem like an odd choice. We have added a short explanation in the introduction on the place physiotherapy private practices have in the Dutch healthcare system, the percentage of physiotherapists that work in this setting, and the specific learning needs. |
| **Abstract**<br>Pg 2, line 23. I don't think there is such as word as fundament- please use another word here | We replaced "fundament" with "foundation". |
| **Introduction**<br>Pg 3, line 17 change 'attention for these factors' to 'attention to these factors' | We have applied your suggested change. |
| Pg 3, line 32 . suggest changing 'hardly any information' to 'a dearth ( or absence or lack) of information' | We have applied your suggested change. |
| Pg 3, line 46 What is CEWIL Canada- provide full word for CEWIL | We have added: "Co-operative Education and Work-Integrated Learning Canada". |
| Pg 3. Line 48 –'Björk & Willermark, 2024'. No need for the year here as you have the numbered citation | We have removed ",2024,". |
| Pg 3, line 49 In relation to this statement 'WIL was unpacked', not 100% clear here- do you mean it was unpacked by the manuscript authors? | We have replaced "unpacked" with "operationalized". |
| Pg 3, lines 57-59. In relation to this sentence 'The group of private practice physiotherapists is not homogenous and will differ in knowledge, skills, attitudes, and experiences'. Some more context is required in relation to the target audience for the WIL. Who are the group? Is if physios in specific region, or nationally? Can you provide some details on profile/ numbers etc. Also suggest remove the word 'will' (future tense). | We have added a short explanation in the introduction on the place physiotherapy private practices have in the Dutch healthcare system, the percentage of physiotherapists that work in this setting, and the specific learning needs.<br><br>We have removed the word "will". |
| Pg 3, lines 60. Can you define who the stakeholders are. By end-users, do you mean people with chronic pain? | Since the aim is to develop a WIL-program, we mean by end-users: the learners. In this context the physiotherapists working with patients with chronic pain. To convey this more clearly, we decided to use the term "target audience" throughout the paper and explained this in the introduction |
| Pg 4, lines 5-6. Please provide a definition of 'co-design' here, as distinct from Design thinking. | We have added the definition of Sanders & Stappers, 2008. |
| **Methods**<br>Pg 5, line 7, Was this specifically for private | |

| | |
|---|---|
| practice physiotherapists in the Netherlands? Please clarify here | We have added the word “Dutch” to clarify that this is indeed developed for physiotherapists in the Netherlands. |
| Pg 5, line 9 suggest minor rewording to ‘The reporting of this study is in accordance with the proposed guideline for REporting Design Research’. Why is it a proposed guideline? | The REDR (REporting Design Research) guideline is mentioned on the EQUATOR Network website. It is described as a: "proposed checklist for reporting on health research involving human-centred design". This means that while REDR is recognized by EQUATOR, it is still considered a proposed guideline. We have added this clarification in the text. |
| Pg 5, line 16. Again, in relation to the larger study, is this specifically targeted towards physiotherapists in the Netherlands? – please clarify | We have added the clarification “Dutch” to clarify the target audience. |
| Pg 5, line 21 this subheading is not clear ‘Participants: core group and stakeholders’, as to what is meant by ‘core group’ as not referred to previously. Regarding stakeholders (and further to my previous comment), did this include the end-users.<br>More clarity is needed in this section regarding the different media used to recruit the different groups, including the ‘experts in chronic pain’ that were mentioned in the introduction. | We have reviewed and amended the terminology and structure of the description of the participants, as well as aligned these more precisely with the figures. |
| Pg 5, line 31<br>The term ‘sprint’ team is used here without out any explanation- please clarify. Suggest explaining earlier in the design section | We have renamed the “sprint team” as “project team”. We have explained the use of the sprint methodology later in the method section and have referred to this section. |
| Description of the various team members is quite confusing to a reader. Whilst table 1 is helpful, it does not really provide information about who occupies these roles. Also, not clear where the end-user features | We have reviewed and amended the terminology and structure of the description of the participants, as well as aligned these more precisely with the figures. We replaced table 1 with a figure (now figure 1) to give a more visual presentation of who occupies which role. |
| Pg 6, lines 16-27 Procedure<br>Whilst figure 1 is helpful, suggest alignment with detail in text regarding participants e.g. patient representative shown in figure not mentioned in the manuscript. Also, phases are described in text format, but not marked in the figure, so suggest adding in to clearly link the figure with the text. | We have further aligned the terminology and marked the phases in the figure (now called figure 2). |
| Pg 6, line 26 does ‘last phase’ refer to phase 4? – please clarify | This does indeed refer to phase 4. We have clarified this in the text. |
| Pg 6, line 45- is the interview guide included as a supplementary file? Suggest it should be | We have added the interview guide in Appendix A and referred to this in the text. |
| Pg 6, line 60- what do you mean by ‘potential attendees’? | We have replaced “potential attendees” with “physiotherapists working in private practice” |
| Pg 7, line 12 – figure 2 does not align with information in text, where phases are described. Can these be merged to show how the Design Thinking elements were incorporated into the different phases described? | Figure 2 shows the Double Diamond. These steps are used within the three design sprints in phase 2. We’ve merged the described steps to fit with the Double Diamond and how we used this in the sprints and aligned the terminology for more clarity. |

| | |
|---|---|
| Pg 7, lines 54-56. How were interview and focus group data collected? E.g. any audio recordings with subsequent transcriptions?<br>Was a specific analytical framework used? What form of analysis was undertaken e.g. thematic analysis? Suggest supporting reference. | Interview and focus group data within phase 2 were collected within MURAL, which is an online whiteboard from the Mural visual collaboration platform. A combination of thematic coding, concept mapping, and relational mapping was employed to synthesize these insights into themes. We have added this explanation and references. |
| Pg 7, line 58- what you mean by 'underlying principles' and provide a supporting reference | By the underlying principles we mean the models and theory that support the different themes that were discussed or uncovered during phase one and 2. We changed the wording to convey this clearer. |
| Suggest moving the ethical considerations up to the beginning of the methods section e.g. design section. | We have moved this to the study design section as per your suggestion. |
| **Results**<br>Pg 8, line 11 delete the year '2021' and change 'focused of' to 'focused on' | <br>We have applied your suggested change. |
| Pg 8, lines 38-39. Not 100% clear what this sentence means 'Interviewees called attention…'. Do you mean 'drew attention' or 'highlighted'? | We have replaced "called attention to" with "highlighted". |
| Pg 8, line 44 what do you mean by 'attention to suitable attitudes'? | We have replaced this by "appropriate professional attitudes". |
| Table 2: Column 1 Treatment 'How might we offer footing in dealing with complexity' not clear what offer footing means? | We have replaced this by "stability". |
| Table 2 Colum 3 Tools what do you mean by journey- ie patient journey? | We mean the learning-journey for the attendees. This has been changed in the text. |
| Figure 3 is very well presented and a strong visual. Glossary is very helpful. There are a couple of minor typographical errors: Collaborative learning and Vygotski board. I don't think 'course-participants' should be hyphenated? Is a course participant the same as an attendee as described in table 2? Suggest use of consistent terms | We have replaced "course-participants" with "attendees".<br><br>We have amended the typographical errors. |
| **Discussion**<br>Pg 13, line 13- where is the role of the end-user here, suggest they could play an important role e.g. development of personas, clinical vignettes? | <br>We have added "target audience" here. |
| Strengths and limitations section- clearly signpost which are the strengths and limitations, suggest placing this section after the comparison with other literature. | We have added signposting and placed the section after the comparison with other literature as suggested. |
| Pg 13, line 36 suggest adding the word 'process' after 'In the co-design sprint' | We have added this per your suggestion. |
| Pg 13, line 40 regarding this statement 'Co-design processes can be fuzzy, lacking clarity when it comes to decision-making', is this your opinion, or can you support with reference? | These challenges are broadly recognised and supported by other authors. See e.g. the references of Antonini, Piirainen, and Thorburn. We have rewritten and extended this section to provide more clarity. |
| In the discussion, can the author team outline next steps? | We have added a section on next steps and recommendations. |
| Some discussion is also warranted on the target audience ie 'physios in private practice' and why/how this will address their learning needs, | We have added the following explanation to the 'Discussion': This design process adopts a work-integrated learning (WIL) approach, in which the |

| | |
|---|---|
| and how they differ to those working in the public system.<br>Was any consideration given to principles of Universal Design for Learning (UDL) https://udlguidelines.cast.org/ | workplace or practice setting plays a central role in the learning process. As the organisational and clinical context of private practice differs from that of the public sector, this WIL programme is specifically designed to meet the needs of physiotherapists working in Dutch private practice settings.<br><br>One of the contributing experts has a background in educational science (specifically: learning environments in vocational education and workplace learning), and their expertise has informed the design of the programme. Based on this expertise attention has been given to 'how humans learn' which is consistent with the UDL framework. |
| Pg 14, lines 16-21.<br>Separate the recommendations and conclusion section. Not clear what the 'recommendations' really refer to. Suggest a more informative subheading | We have separated the recommendations and conclusions section and expanded the recommendations section with 'next steps'. |
| Conclusion should provide a brief summary of the aims, methods and results. Currently, it does not really conclude the key points. | We have rewritten the 'conclusions' section and added a summary as per your suggestion. |
| | |
| Reviewer: 2 | |
| Explain in the details the validity of this program. Why only private practice was selected. | We have added a short description of the Dutch setting, explaining how this choice is valid for the Dutch context, including why private practice was selected. |
| Describe the in details, what were limitations faced during the study. | We have expanded the strengths and limitations subsection in the discussion section. |
| Explain and brief the questionnaire used during the study. | We have added the interview guide used in Appendix A and referred to this in the text. |
| Adress the validity and reliability of the presented co-design. | We have addressed the validity and reliability question in the 'strength and limitations' subsection in the discussion. |

## VERSION 2 - REVIEW

**Reviewer** 1

**Name** French, Helen

**Affiliation** Royal College of Surgeons in Ireland, School of Physiotherapy

**Date** 10-Aug-2025

**COI**

Many thanks to the author team for addressing the feedback. The revised version of the manuscript is much clearer as a result. Some very minor comments below ( based on clean copy of revision)

Abstract

Page 2. Strengths and limitations not clearly delineated

Introduction

Page 4, line 20, (newly added text)- Suggest remove the wording ‘In specific’

Methods

Page 5, line 52. Was the student a physiotherapy student? Please clarify

Page 6, line 26. Suggest minor rewording to newly inserted text to ‘to also test

and give feedback on the prototypes’.

Discussion

Page 13, line 50. Suggest changing ‘hereby’ to ‘thereby’

Page 14, line 7-8 (newly inserted text) ‘gaps can lead to blind sports and

reduce the comprehensiveness of the design’ Typing error here? Should this be ‘blind spots’. Language a little colloquial and lacks clarity, so could you reword ‘blind spots’?

Figure 1

Typing error in ‘validation sessions’- i is missing

---

## VERSION 2 - AUTHOR RESPONSE

| Reviewer: 1 | |
|---|---|
| **Abstract** | |
| Page 2. Strengths and limitations not clearly delineated | We have revised the section to clearly separate the strengths and limitations of the study. The strengths are now listed under a dedicated heading, followed by a distinct paragraph outlining the limitation. We have slightly expanded the description of the limitation for more clarity. |
| **Introduction** | |
| Page 4, line 20, (newly added text)- Suggest remove the wording ‘In specific’ | We have removed the wording ‘In specific’ as per your suggestion. |
| **Methods** | |
| Page 5, line 52. Was the student a physiotherapy student? Please clarify | The student mentioned was indeed a physiotherapy student. We have clarified this in the text. |

| | |
|---|---|
| Page 6, line 26. Suggest minor rewording to newly inserted text to ‘to also test and give feedback on the prototypes’. | We have changed the text as per your suggestion. |
| **Discussion** | |
| Page 13, line 50. Suggest changing ‘hereby’ to ‘thereby’ | We have changed the text as per your suggestion. |
| Page 14, line 7-8 (newly inserted text) ‘gaps can lead to blind sports and reduce the comprehensiveness of the design’ Typing error here? Should this be ‘blind spots’. Language a little colloquial and lacks clarity, so could you reword ‘blind spots’? | This is indeed a typing error and was meant to be ‘blind spots’. As per your suggestion to be less colloquial and more clear, we have reworded the sentence to: ‘Such gaps may lead to omissions in the design process, thereby limiting its overall comprehensiveness.’ |
| **Figure 1** | |
| Typing error in ‘validation sessions’- i is missing | Thank you for pointing this out. With the addition of a new figure for the participants being figure 1, I understand this comment to refer to the project journey (now figure 2).<br><br>We have amended the typing error. |
| | |
